# Childhood Sexual Behavior as a Mediator of the Relationship Between Sexual Abuse and Early Sexual Debut: A Prospective Analysis

**DOI:** 10.1007/s10508-025-03228-w

**Published:** 2025-08-05

**Authors:** Brian Allen

**Affiliations:** 1 Departments of Pediatrics and Psychiatry, Penn State College of Medicine, Hershey, PA USA; 2 Center for the Protection of Children, Penn State Health Children’s Hospital, 500 University Drive, Penn State Hershey Medical Center, Hershey, PA 17033 USA

**Keywords:** Sexual debut, Child sexual behavior, Sexual preoccupation, Sexual abuse, Physical abuse

## Abstract

Childhood sexual abuse (CSA) is a commonly recognized risk factor for early sexual debut. However, relatively few studies are available that examine the potential mediators of this relationship. One promising marker is a preoccupation with sex and sexual topics as one enters the teenage years; however, sexual behavior observed at age 8 might serve as an even earlier risk indicator for early sexual debut. This study used the prospective data of 697 female children to test a serial multiple mediation model where sexual abuse predicts sexualized behavior at age 8, which in turn predicts sexual preoccupation at age 12, and this sequence then forecasts engaging in sexual intercourse at age 14. Given that child physical abuse (CPA) is also commonly linked to the display of childhood sexualized behavior, this variable was included in the models. Path analyses determined that the best-fitting model included sexual behavior as measured at age 8 and sexual preoccupation measured at age 12 in a serial mediator model, which effectively accounted for the relationship between early CPA and CSA and early sexual debut. Contrary to expectations, CPA emerged as an independent predictor of sexual behavior while CSA did not after the effects of CPA were controlled. The implications of these findings for understanding risk for early sexual intercourse are discussed, including treatment/prevention suggestions.

## Introduction

Current estimates are that approximately 18% of female children worldwide experience child sexual abuse (CSA; Stoltenborgh et al., 2011). The short and long-term consequences of CSA are well-established and include myriad emotional and behavioral problems (Hailes et al., 2019; Lindert et al., 2014). Untoward sexual outcomes have long been recognized as common consequences of CSA (e.g., Beitchman et al., 1991), including an earlier onset of sexual debut (Miller et al., 1995; Noll et al., 2003), which subsequently increases the youth’s risk for teen pregnancy (Noll et al., 2009) and contracting a sexually transmitted infection such as HIV/AIDS (Centers for Disease Control & Prevention, 2007). Although defining the age at which voluntary sexual intercourse might be considered “early” or “risky” is fairly arbitrary and culturally dependent, a number of researchers have suggested that the age of 14 years serves as a useful benchmark (e.g., Asare et al., 2023; Jones et al., 2010; Wilson & Widom, 2008; Zimmer-Gembeck & Helfand, 2008).

Of course, many children who experience CSA do not engage in early sexual intercourse or other types of sexual risk-taking. This prompted researchers to search for potential mediating factors to allow for early identification of those most at risk. Multiple studies have investigated sexual preoccupation as one such risk factor. Sexual preoccupation is typically defined as repeatedly thinking about sexual topics and/or performing behaviors that suggest the individual is ruminating on sexual topics (e.g., viewing pornography, frequent masturbation, talking in a sexual manner). Noll et al. (2011) tested a model with a sample of adolescent girls where sexual preoccupation was hypothesized to mediate the relationship between child maltreatment, including sexual abuse, and risky sexual behaviors. Results indicated that sexual preoccupation was a stronger mediator of the relationship than behavioral problems, parent–child relationship, associating with a risky peer group, and substance use. Simon and Feiring (2008) analyzed a longitudinal dataset that followed children beginning at the point of disclosure of sexual abuse (ages 8–15). Results suggested that those youth with greater levels of sexual preoccupation at disclosure and approximately 1-year post-disclosure were at increased risk of various sexual risk-taking behaviors at a 6-year follow-up assessment.

Although sexual preoccupation appears to be a plausible mediator of the relationship between sexual abuse and risky sexual behavior, including early sexual intercourse, measures of sexual preoccupation typically rely on the youth having sufficient cognitive ability to self-report their thoughts and feelings. For instance, in both the Noll et al. (2011) and Simon and Feiring (2008) studies, the children enrolled in the study and completing the measures mostly were in the pre-teen (ages 10–12) or early teenage years (ages 13–15). This limitation restricts the use of sexual preoccupation as a risk factor to older children capable of self-reporting and, therefore, on the verge of entering the adolescent years. Identifying an earlier mediator of the relationship between CSA and early sexual intercourse, or an earlier precursor to sexual preoccupation, may allow for targeted treatment of concerns before children reach the age at which sexual intercourse is more likely. However, most studies examining the development of sexual preoccupation have focused exclusively on establishing CSA as an etiological factor.

One study, however, did examine a construct that may be more useful for studying earlier ages. Negriff (2018) examined willful and observable sexual behavior starting at the age of 10 (mean age, 10.98 years) in a longitudinal, four-wave study followed through the age of 18 (mean age, 18.22 years). She found that sexual behavior at each timepoint predicted the display of sexual behavior at the next timepoint throughout the duration of the study. However, whether sexual behavior is linked to sexual preoccupation is unclear. Only two studies were identified that examined the connection between these two variables. Noll, et al. (2003) assessed the relationship between sexual behavior in a group of youth (mean age, 11.11 years) to sexual preoccupation as reported approximately 9 years later. The resulting model, which included several other variables, failed to establish a connection between sexual behavior and later sexual preoccupation. Friedrich et al. (1997) reported a concurrent correlation between parent-reported sexual behaviors and child self-reported sexual concerns, a broader concept that includes sexual preoccupation and anxiety about sexual topics. They noted a strong correlation in their sample of 83 children ages 7–12, but the result was not statistically significant. These results appear to suggest that a connection between sexual behaviors and sexual preoccupation is unlikely; however, the age ranges of samples were wide and the temporal association between the measures was either simultaneous or quite prolonged.

Theoretically, studies examining the link between CSA and the development of childhood sexual behavior and/or preoccupation commonly rely on the traumatic sexualization hypothesis of Finkelhor and Browne (1985). This theory suggests that CSA results in ongoing sexual concerns through two primary mechanisms. First, CSA provides a social learning experience where the child develops the perspective that sex is a functional or expected behavior. This may manifest in numerous ways. For instance, the child may begin behaving in a sexualized way to gain attention or gifts, or the child may develop maladaptive beliefs that sex is a common activity between individuals. Second, the experience of CSA may prompt posttraumatic stress symptoms where the child experiences intrusive thoughts of a sexual nature and/or experiences physiological hyperarousal that increases the likelihood of acting on sexual thoughts and feelings. Empirical findings testing this theory are mixed. While there is considerable evidence that CSA predicts the display of sexual behavior and preoccupation (e.g., Allen et al., 2025; Noll et al., 2011), there is less evidence that sexual abuse-related PTS is uniquely associated with childhood sexual behavior (Allen, 2023a). The conclusion from much of this latter work is that social learning may be the operative factor in explaining the etiological role of CSA in the development of sexual behavior.

The current study tested a number of hypotheses and questions. First, in an attempt to extend the findings from Negriff (2018) to a younger age, it was hypothesized that caregiver-reported sexual behavior assessed at age 8 would significantly mediate the relationship between CSA and early sexual intercourse (i.e., by the age of 14). Second, in an attempt to link the literature on sexual behavior and sexual preoccupation, it was hypothesized that child sexual behavior at age 8 would predict one’s level of sexual preoccupation at age 12, and that sexual preoccupation would in turn predict early sexual intercourse. In this manner, age 8 sexual behavior is hypothesized to not only function as an independent mediator of the relationship between CSA and early sexual intercourse, but also serve as an early precursor to age 12 self-reported sexual preoccupation. In recognition of temporal proximity, it was further hypothesized that the serial multiple mediator model, including both age 8 sexual behavior and age 12 sexual preoccupation, would perform better in explaining the data than the model including only age 8 child sexual behavior.

Two specific methodological issues were identified and addressed during the design of the current study. First, there is an obvious phenomenological connection in the relationship between CSA, child sexual behavior, sexual preoccupation, and early sexual intercourse. However, studies demonstrate that non-CSA forms of maltreatment also are related to early sexual intercourse (Negriff et al., 2015; Noll & Shenk, 2013). In addition, various forms of maltreatment are connected to the display of childhood sexual behavior, including exposure to inter-partner violence, (Cale & Lussier, 2017), neglect and psychological maltreatment (Merrick et al., 2008), and physical abuse (Allen, 2017), as well as various forms of parental factors (see Allen, 2023b for a review). In a recent meta-analysis, physical abuse was the one form of maltreatment identified as having the clearest link to PSB, after CSA, with a moderate size effect (Allen et al., 2025). Indeed, some evidence suggests that the experience of physical abuse may be more influential for the emergence of problematic sexual behavior than CSA (Allen, 2023a; Friedrich et al., 2003). With this background, child physical abuse was also included in the tested model.

Second, studies repeatedly demonstrate that female adolescents are significantly less likely to report sexual preoccupation or engage in consensual sexual activity than their male counterparts (Kushal et al., 2022; Nilsson et al., 2008; Reis et al., 2024). In addition, the study by Noll et al. (2011) included only female adolescents, while the Simon and Feiring (2008) study included a significant proportion of female participants (76%). There is also evidence that gender exhibits a small, though significant, relationship with childhood sexual behavior (Allen et al., 2025). As such, the current model tested here was developed largely based on research and findings that are female-centric. As such, it was deemed appropriate to test the model with female participants.

## Method

### Participants

Data for the current study were drawn from the Longitudinal Studies in Child Abuse and Neglect (LONGSCAN) consortium. LONGSCAN was a collaboration of researchers at 5 sites across the United States each conducting individual prospective studies examining the developmental impact of child maltreatment. To promote cross-site pooling of relevant data, the sites coordinated the use of a common set of measures, assessment time points (every two years), and research procedures, although each site remained unique in other aspects depending on the specific goal of the study. The specific composition of each sample differed based on site; however, each site included a sample of maltreated children and a control group drawn from a socioeconomic risk category (e.g., low socioeconomic status, urban areas). A total of 697 female children and their caregivers were enrolled in the study at the age 4 assessment, which marked the collective beginning of the project (some sites had collected data prior to age 4). The current study made use of the data collected at the age 8, 12, and 14 assessments. Although the total sample was fairly evenly distributed across the five sites, the majority of the sample identified as Black/African-American (see Table 1). Data collection at all LONGSCAN sites was approved by applicable Institutional Review Boards, as was this secondary data analysis. All participants provided informed consent prior to participation in LONGSCAN. For more detailed information about LONGSCAN, the reader is referred to Runyan et al. (1998).

**Table 1:** Demographic information and descriptive statistics

Variable	*n*(*%*)	*M*(*SD*)
LONGSCAN site		
East	135 (19.4)	
Midwest	130 (18.7)	
Northwest	125 (17.9)	
South	133 (19.1)	
Southwest	174 (25.0)	
Ethnicity		
Black/African-American	381 (54.7)	
White	177 (25.4)	
Mixed ethnicity	80 (11.5)	
Other ethnicities	59 (8.5)	
Age of Child at Assessment		
Age 8		8.3 (.43)
Age 12		12.4 (.46)
Age 14		14.3 (.46)
Child Sexual Behavior Inventory raw score (age 8)		2.5 (3.61)^a^
TSCC-Sexual Preoccupation *T*-score (age 12)		50.3 (16.09)^b^
Have ever had sexual intercourse (age 14)		
Yes	65 (9.3)	
No	362 (51.9)	
Missing	270 (38.7)	

TSCC, Trauma Symptom Checklist for Children, ^a^Missing n = 143, ^b^Missing n = 274.

### Measures

*Child Maltreatment Reports*: LONGSCAN investigators reviewed and coded Child Protective Services (CPS) records using the Modified Maltreatment Classification System (MMCS; English and the LONGSCAN Investigators, 1997; as modified from the Maltreatment Classification System of Barnett et al., 1993). For the current study children were classified as experiencing a specific type of abuse if an allegation of such abuse was present in the CPS record at the time of the age 8 assessment. Records were available for all 697 cases. Sexual abuse was alleged for 118 of the children (16.9%) and physical abuse was alleged in 182 cases (26.1%), with 63 children having allegations of both types of abuse (9%). All coders of CPS records were trained to standard by LONGSCAN investigators. Inter-rater reliability (kappa) for the sexual abuse allegations was calculated as 0.77 and physical abuse allegations as 0.87 (English and the LONGSCAN Investigators, 1997).

*Child Sexual Behavior*: The Child Sexual Behavior Inventory (CSBI) assesses the frequency of various child sexual behaviors (Friedrich, 1997). Caregivers rate the frequency of each behavior on a scale ranging from 0 (never occurred in the past 6 months) to 3 (occurred at least once per week in the past 6 months). The LONGSCAN investigators consulted with Friedrich to trim the original 36-item CSBI to a 26-item version that was then included in the LONGSCAN studies at the age 8 assessment (example items: “Tries to undress others against their will,” “Shows private parts to children,” “Talks about sexual acts”). This was done to shorten the time required and to remove items that might most be viewed as objectionable by participating caregivers (e.g., “Puts objects in vagina or rectum”). The original CSBI was validated with a sample of over 1,100 children (Friedrich et al., 1992). This abbreviated version of the CSBI has been used in several other analyses of the LONGSCAN data (e.g., Allen, 2017; Wamser-Nanney & Campbell, 2019) and found to relate to other constructs as expected. For the current study, child sexual behavior was measured by summing the scores for each of the 26 items to arrive at a total raw score (Cronbach’s α = 0.74). Because *T*-scores were not available and the measure is significantly shorter than the full measure, no attempt is made to determine the point at which sexual behavior is of a problematic or “clinical” nature. Rather, sexual behavior is viewed as a continuum with higher scores suggesting a greater frequency of displaying various sexual behaviors.

*Sexual Preoccupation*: Briere (1996) developed the Trauma Symptom Checklist for Children (TSCC) to assess self-reported emotional and trauma-related concerns of children and adolescents between the ages of 8 and 16. The child is presented with 54 symptoms or problems and asked to report the frequency with which she experiences each on a scale from 0 (Never) to 3 (Almost all the time). The TSCC was standardized on a large sample of children and displays acceptable psychometric properties (Briere, 1996). The current study uses the Sexual Preoccupation scale of the TSCC administered at the age 12 assessment. This scale includes 7 items assessing repetitive sexual thoughts, desire to engage in sex, sexual physiological arousal, and frequency of self-stimulation (example items: “Thinking about having sex,” “Having sex feelings in my body,” “Touching my private parts too much”). Scores for the items are summed and converted to age and gender-referenced T-scores (Cronbach’s α = 0.79). As a data check, scores on this scale were contrasted between those who reported having already experienced menarche at the age 12 assessment (56.3%; *M* = 50.36, *SD* = 13.85) and those who had not (*M* = 49.93, *SD* = 15.23); there was not a significant difference (*t* = 0.30, *p* = 0.765).

*Early Sexual Debut*: The LONGSCAN researchers developed an adolescent sexual experiences measure that was administered at the age 14 assessment (Knight et al., 2008). At the beginning of the measure adolescents were asked, “Have you ever had sex?” An additional statement clarified that this question referred specifically to sexual intercourse, although the term “intercourse” was not clearly defined. Of the 697 female participants in the sample, 65 provided a positive endorsement (coded 0 = no; 1 = yes), while the data was missing for 270 (note that adolescents responding “I don’t know” to this question were classified as “missing”). Of these 65 adolescents, a review of the CPS records showed that 11 had a sexual abuse experience prior to the age of 14 that included some form of sexual intercourse (i.e., oral, anal, vaginal). This is potentially problematic as adolescents may have reported their sexual abuse as early sexual intercourse, thus confounding results. However, a chi-square analysis of all teens with a sexual abuse history suggested no difference in the frequency with which they endorsed having sexual intercourse at age 14 based upon whether their prior sexual abuse experiences included intercourse (*χ*^*2*^ = 0.122, *p* = 0.73). In addition, the vast majority of the sample (96.4%) had experienced menarche by the age 14 assessment, and this status was not related to reports of early sexual debut (*χ*^*2*^ = 2.80, *p* = 0.094).

### Data Analyses

A series of path analyses were conducted to examine the hypotheses. The first model tested the first hypothesis that sexual behavior at age 8 would serve as a significant mediator and, thus, risk indicator of early sexual intercourse. The second model tested the model established in previous research where sexual preoccupation is the sole mediator. The third model tested a serial mediator model where the impact of sexual abuse on early sexual intercourse is believed to operate through sexual behavior at age 8 and then sexual preoccupation at age 12. To allow for a direct comparison between the models, the simpler models were nested within the larger serial mediator model and a change in chi-square test was performed. These analyses used all 697 female participants in the dataset. Physical abuse history was entered as a predictor maltreatment variable in all models. Recruitment site (0 = mixed sampling methods; 1 = Samples only referred by Child Protective Services) was controlled in all analyses as is standard practice in LONGSCAN research (e.g., Black et al., 2009; Merrick et al., 2008). All path analyses were modeled using Mplus 8.11 (Muthen & Muthen, 2024).

Maximum likelihood estimation was used to account for the missing data. However, because analyses suggested that the continuous mediating variables (i.e., CSBI, TSCC-Preoccupation) were significantly positively skewed, maximum likelihood estimation with robust standard errors (MLR) was employed. In addition, since sexual intercourse at age 14 is a dichotomous variable, Monte Carlo numerical integration was used in conjunction with MLR estimation. MLR estimation uses logistic regression to model categorical dependent variables, and odds ratios are provided for those pathways (denoted in the figures using italic font). Model fit indices are not applicable in cases of dichotomous outcomes using MLR; therefore, interpretation of statistical analyses focused on the strength of the path coefficients obtained. For paths toward continuous mediator variables the unstandardized beta coefficient (*b*) is reported. As a result of fit indices not being available for these path analyses, change in chi-square tests comparing models utilized a modified form of the Satorra-Bentler scaled chi-square applicable to nested models (Satorra, 2000; formulae for calculations available at http://www.statmodel.com/chidiff.shtml). When results indicated a significant difference between models, the model with the lower Akaike Information Criterion (AIC) was retained.

## Results

### Single Mediator Models

**Fig. 1:**
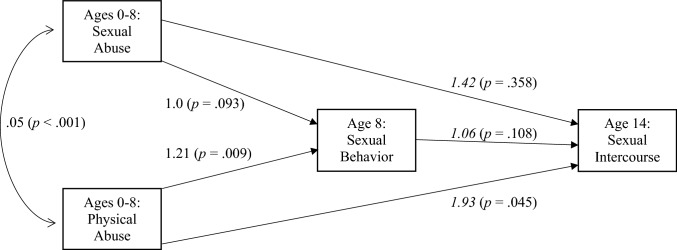
Model 1 predicting the impact of child abuse on sexual intercourse by age 14, child sexual behavior as a mediator. Plain font is unstandardized beta coefficients (*b*), italic font is odd ratios. *n* = 697

As shown in Fig. 1, the first model examining the role of child sexual and physical abuse on early sexual intercourse failed to find that increased sexualized behavior at age 8 predicted early sexual intercourse. It should be noted, however, that the role of physical abuse appeared prominent as it independently predicted both sexual behavior at age 8 and early sexual intercourse at age 14 after controlling for sexual abuse. Although sexual abuse was related to age 8 sexual behavior using bivariate correlations (*r* = 0.15, *p* < 0.001; see Table 2), such a relationship failed to reach the customary level of statistical significance once physical abuse was controlled in the equation. Interestingly, sexual abuse increased the individual’s risk of early sexual intercourse by 42%, but this finding was not statistically significant as a result of the variability observed.

**Table 2:** Zero-order correlations

	1	2	3	4	5
1. Sexual abuse experience^#^ (prior to age 8)	–				
2. Physical abuse experience^#^ (prior to age 8)	.28^***^	–			
3. Child Sexual Behavior Inventory raw scores (age 8)	.15^***^	.18^***^	–		
4. TSCC-Sexual Preoccupation *T*-scores (age 12)	.07	.06	.15^**^	–	
5. Early sexual intercourse^#^ (age 14)	.07	.09	.10^*^	.33^***^	–

^*^p < .05, ^**^p < .01, ^**^^*^p < .001. Items denoted with a “^#^” were dichotomized.

**Fig. 2:**
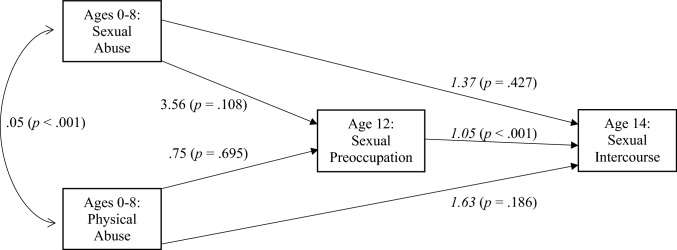
Model 2 predicting the impact of child abuse on sexual intercourse by age 14, sexual preoccupation as a mediator. Plain font is unstandardized beta coefficients (*b*), italic font is odd ratios. *n* = 697

Figure 2 shows results for the model previously established in the research; sexual preoccupation at age 12 is the sole mediator of the relationship between child abuse and early sexual intercourse. In this model age 12 sexual preoccupation is significantly related to early sexual intercourse; however, neither form of child abuse appears related to the mediator or the outcome. Again, the impact of sexual abuse on both age 12 sexual preoccupation and early intercourse appears large, but the variability involved prevented the relationships from reaching statistical significance.

### Multiple Serial Mediator Model

**Fig. 3:**
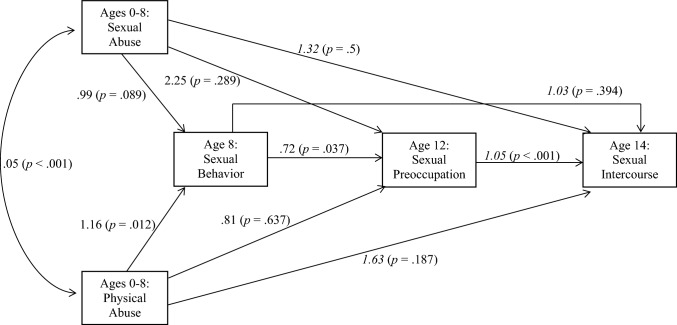
Model 3 predicting the impact of child abuse on sexual intercourse by age 14, serial mediator model. Plain font is unstandardized beta coefficients (*b*), italic font is odd ratios. *n* = 697

The serial mediator model predicting early sexual intercourse (Fig. 3) provided multiple interesting results. First, the Satorra-Bentler scaled chi-square difference was calculated to examine whether the serial mediator model was preferred over each of the nested single mediator models. In comparison to Model 1 that included only age 8 sexual behavior as a mediator, the serial mediator model was significantly different (*χ*^*2*^ = 26.47, *p* < 0.001) and the AIC preferred the serial model. Likewise, the serial mediator model demonstrated a lower AIC than the nested model including only age 12 sexual preoccupation as the sole mediator and these two models were significantly different (*χ*^*2*^ = 19.71, *p* < 0.001). Consequently, the multiple serial mediator model including both age 8 sexual behavior and age 12 sexual preoccupation emerged as the preferred model.

Examining the functioning of the serial mediator model showed that sexual behavior at age 8, indeed, predicted sexual preoccupation at age 12, which then predicted a greater likelihood of having had sexual intercourse by age 14. Each one-point increase in sexual preoccupation at age 12 increased one’s risk for early sexual intercourse by 5%. Although this may seem small, one must consider that the average standard deviation for a *T*-score is 10 points. Therefore, individual’s one standard deviation above the mean of 50 at 12 years of age were 50% more likely to engage in early sexual intercourse by age 14; individuals at the clinical cutoff score of 70 at age 12 were twice as likely as an individual scoring at the mean to engage in sexual intercourse by age 14. Lastly, this model suggests that neither form of child abuse directly impacted sexual preoccupation or early sexual intercourse, but physical abuse exerted an indirect effect through increasing the score for sexual behavior at age 8. It is notable that this finding controlled for the influence of sexual abuse. Sexual abuse, on the other hand, did not exert a significant effect after controlling for physical abuse.

## Discussion

This study sought to determine whether childhood sexual behavior was a significant mediator of the relationship between child abuse and early sexual debut, and/or a precursor of increased sexual preoccupation, which was previously identified as a mediator of early sexual debut. The first hypothesis, that child sexual behavior would directly mediate the relationship between child physical and/or sexual abuse and early sexual intercourse, was not supported as a direct link was not observed between child sexual behavior and early sexual intercourse. However, the second hypothesis was supported as childhood sexual behavior mediated the relationship between physical abuse and sexual preoccupation at age 12. Sexual preoccupation, in turn, predicted sexual intercourse by age 14. As such, child sexual behavior assessed at age 8 appears best conceptualized as an early indicator of future sexual preoccupation and that this rumination on sex and sexual topics places a child at increased risk of early sexual intercourse. In addition, results favored this model over another model that omitted child sexual behaviors and included only sexual preoccupation as a mediator of the relationship between child sexual and physical abuse and early sexual intercourse.

These results do not appear to support the traumatic sexualization theory (Finkelhor & Browne, 1985), as the experience of physical abuse remained significant in the model, which effectively controlled for sexual abuse. After controlling for the impact of physical abuse, sexual abuse was not a significant predictor of the mediating variables or the early sexual intercourse outcome. The pathways identified in the retained model (Fig. 3) suggest a sort of sexualized developmental cascade effect where physical abuse prior to age 8 prompts a greater display of sexualized behaviors at age 8, leading to an increased likelihood of sexual thoughts and arousal, which then predisposes a child to engage in early sexual behavior. Caution is urged, however, in conceptualizing child sexual behavior and sexual preoccupation as distinct phenomena. The sexual behavior observed by the caregivers at the age 8 assessment may be the outward manifestations of sexual preoccupation on the part of the child, which would suggest that the relationship with the age 12 subjective report of the child is a matter of construct stability and cross-reporter agreement. Nonetheless, the results do provide evidence that sexualized behaviors at age 8 predict self-reported sexual preoccupation at age 12, which in turn predicted sexual intercourse by age 14.

A developmental psychopathology approach can help explain the connection between physical abuse and the emergence of problematic sexual behavior (Allen, 2023b; Elkovitch et al., 2009). Problematic sexual behavior of children typically correlates with other internalizing and externalizing problems (Allen, 2023a; Allen et al., 2015; Friedrich et al., 2003), suggesting that children displaying increased levels of sexual behavior may experience problems with core regulatory capabilities (e.g., emotion dysregulation, poor social skills, behavior dysregulation). The experience of child maltreatment is consistently linked to the poor development of these social-emotional skills (Alink et al., 2009; Kim-Spoon et al., 2013). In a direct test of this proposition, Allen (2023a), using a different dataset, found that physical abuse predicted problems with self-regulation after controlling for sexual abuse, but sexual abuse did not predict such problems after controlling for physical abuse. In this manner, increased sexual behavior, including early sexual debut, can be conceptualized as a manifestation of core deficits that may emerge as a result of physical abuse or other types of maltreatment, much as how many other forms of child psychopathology may emerge from maltreatment.

It has been postulated that the observed differential impact of the two types of abuse may be the result of variance in severity and frequency (see Allen, 2023b; Friedrich et al., 2003). In effect, non-invasive and singular incidents of sexual abuse are likely to be investigated, documented in records, and reported on questionnaires when they become known, but singular and minor acts of physical abuse are not likely to be treated in the same way. Indeed, state regulations often require that physical discipline reaches a threshold of severity before being considered abusive, while sexual abuse is more commonly defined by the act being committed as opposed to a threshold of severity being reached. As such, documented acts of physical abuse typically involve a certain level of harshness that may logically be presumed to negatively impact development while the same cannot be said for documented acts of sexual abuse. This could also explain why the current study documented a strong, but non-significant, effect size between sexual abuse and several variables; the impact of sexual abuse on other variables fluctuated widely from person-to-person and the between-subjects error prevented a significant statistical result.

Taking a developmental psychopathology approach, an intervention focused on improving broader child behavioral and emotional concerns may be appropriate. Parent–Child Interaction Therapy (PCIT), for instance, has demonstrated efficacy for improving parenting abilities and externalizing concerns among maltreated children (Chaffin et al., 2004; Timmer et al., 2010) and some evidence suggests effectiveness for reducing sexual concerns among children as well (Allen et al., 2016; Friedrich, 2007). In addition, evidence is accumulating that sexual behaviors may be treated with a cognitive-behavioral model that teaches caregivers behavior management skills and focuses on the development of coping skills and sexual awareness among children (Allen et al., 2018, 2025; Bonner et al., 1999; Silovksy et al., 2007). Although it is a defensible hypothesis that these interventions may correspondingly reduce the risk of early sexual debut, the effectiveness of these models for this purpose is not established.

The results of this study should be considered in the context of the limitations present. First, although the CSBI is commonly employed for the assessment of sexual behaviors in children, the measure used in LONGSCAN was a modified version of the measure. As such, several items were omitted and *T*-scores were not available. In addition, the more severe items of the CSBI, including items related to penetrative forms of behavior, were excluded and this prevented determining the relationship of these behaviors to later endorsement of sexual intercourse. Second, the measure of early sexual intercourse was a single item that relied on self-reports of the children that could not be verified. In addition, the item referred specifically to sexual intercourse, which is poorly defined. It may also be helpful to broaden the question to look at voluntary sexual contact of any sort, regardless of whether intercourse occurred. Similarly, identification of sexual and physical abuse relied on allegations documented in CPS records. There is some evidence that this is an underestimate of the true prevalence of abuse and other methods (e.g., interview, self-report) may yield different findings (Cooley et al., 2022). Third, the study used an exclusively female sample. This is appropriate given that the research on which the model was constructed was overwhelming female, but it should not be assumed that these results generalize to male youth. Lastly, future studies should examine longer term and more varied outcomes. Although sexual intercourse by age 14 is an important consideration, sexual risk-taking behaviors (e.g., number of partners, use of contraception) may be more potent predictors of untoward consequences such as pregnancy and sexually transmitted infections. To answer these questions, it may be more beneficial to examine children later in the teenage years where a greater proportion of youth would report having sexual experiences.

In addition to research addressing these limitations, other avenues appear particularly relevant. First, the maltreatment experiences examined in this study were treated as dichotomous variables, which may serve to obscure important differences in abusive experiences. Studies have examined the impact of factors such as the severity, frequency, and chronicity of abuse experiences on later outcomes, often with mixed results. Nonetheless, the role of these characteristics of abuse experiences should be investigated to determine the type of experience necessary to prompt later sexual behavior, sexual preoccupation, and early sexual intercourse. Second, it should be noted that the impact of sexual abuse and physical abuse were considered separately in this study to specifically examine whether pathways focused on sexual behavior and sexual preoccupation required a previous experience of sexual abuse to emerge as predictors of early sexual intercourse. However, as found in this study, various forms of maltreatment and trauma are highly correlated and research in recent years demonstrates that polyvictimization may be a more potent predictor of emotional and behavioral concerns than a single form of trauma considered in isolation (Finklelhor et al., 2007; Layne et al., 2014). The impact of polyvictimization on early sexual intercourse deserves attention.

In summary, the model tested in this paper suggests that sexual behavior observed as young as age 8 may serve as a risk factor for future early sexual debut among females, primarily through its ability to identify children who are most likely to report sexual preoccupation at age 12. This not only proposes an avenue through which to decrease the risk of later untoward sexual outcomes by identifying young children displaying such a propensity, but simultaneously underscores the need to ensure that young children displaying problematic forms of sexual behavior receive effective intervention. Although further research is needed to test the model and other factors that may be operative, the current study suggests there is benefit in looking for the roots of early sexual debut in childhood.
